# Single-molecule sequencing reveals the molecular basis of multidrug-resistance in ST772 methicillin-resistant *Staphylococcus aureus*

**DOI:** 10.1186/s12864-015-1599-9

**Published:** 2015-05-16

**Authors:** Eike J Steinig, Patiyan Andersson, Simon R Harris, Derek S Sarovich, Anand Manoharan, Paul Coupland, Matthew TG Holden, Julian Parkhill, Stephen D Bentley, D Ashley Robinson, Steven YC Tong

**Affiliations:** Menzies School of Health Research, Darwin, Northern Territory Australia; Wellcome Trust Sanger Institute, Wellcome Trust Genome Campus, Hinxton, Cambridge, UK; Pushpagiri Research Center, Pushpagiri Institute of Medical Sciences and Research Center, Thiruvalla, India; School of Medicine, University of St. Andrews, St. Andrews, UK; Department of Microbiology, University of Mississippi Medical Center, Jackson, MS USA

**Keywords:** *Staphylococcus aureus*, MRSA, ST772, Antibiotic resistance, Mobile genetic elements, Complete genome, DAR4145, India

## Abstract

**Background:**

Methicillin-resistant *Staphylococcus aureus* (MRSA) is a major cause of hospital-associated infection, but there is growing awareness of the emergence of multidrug-resistant lineages in community settings around the world. One such lineage is ST772-MRSA-V, which has disseminated globally and is increasingly prevalent in India. Here, we present the complete genome sequence of DAR4145, a strain of the ST772-MRSA-V lineage from India, and investigate its genomic characteristics in regards to antibiotic resistance and virulence factors.

**Results:**

Sequencing using single-molecule real-time technology resulted in the assembly of a single continuous chromosomal sequence, which was error-corrected, annotated and compared to nine draft genome assemblies of ST772-MRSA-V from Australia, Malaysia and India. We discovered numerous and redundant resistance genes associated with mobile genetic elements (MGEs) and known core genome mutations that explain the highly antibiotic resistant phenotype of DAR4145. Staphylococcal toxins and superantigens, including the leukotoxin Panton-Valentinin Leukocidin, were predominantly associated with genomic islands and the phage φ-IND772PVL. Some of these mobile resistance and virulence factors were variably present in other strains of the ST772-MRSA-V lineage.

**Conclusions:**

The genomic characteristics presented here emphasize the contribution of MGEs to the emergence of multidrug-resistant and highly virulent strains of community-associated MRSA. Antibiotic resistance was further augmented by chromosomal mutations and redundancy of resistance genes. The complete genome of DAR4145 provides a valuable resource for future investigations into the global dissemination and phylogeography of ST772-MRSA-V.

**Electronic supplementary material:**

The online version of this article (doi:10.1186/s12864-015-1599-9) contains supplementary material, which is available to authorized users.

## Background

*Staphylococcus aureus* is one of the leading causes of hospital-associated infections worldwide, with clinical manifestations including skin and soft-tissue infections, sepsis, pneumonia and toxic shock syndrome. A large proportion of these infections can be attributed to globally disseminated, methicillin-resistant clones associated with healthcare settings (HA-MRSA), often arising in individuals with predisposing risk factors [1]. In the past two decades, community-associated methicillin-resistant *S. aureus* (CA-MRSA) clones distinct from HA-MRSA clones have emerged to cause infections in otherwise healthy individuals and are often characterised by enhanced virulence and transmissibility [2]. Some of these clones have become a major cause of nosocomial infections, obscuring the distinction between CA-MRSA and HA-MRSA [3-5]. Although most research has so far originated from industrialised countries such as the United Kingdom and United States [2], there is an increasing awareness of the emergence of CA-MRSA clones in non- and newly-industrialised nations in Africa, Asia and the Indian subcontinent [6-11]. Some of these clones have now become globally disseminated [2,10].

Sequence type 772 (ST772) was originally isolated from India and Bangladesh, where it has become increasingly common [7,9,12-14] and appears capable of invading healthcare environments and displacing previously successful nosocomial MRSA [7,15]. It has subsequently been identified in England, France, Germany, Ireland, Italy, Norway, Abu Dhabi, Saudi-Arabia, Malaysia, Australia and New Zealand [8], often in patients with family background or travel histories to India or Bangladesh [15-18]. In the past few years, several draft genomes have been assembled, revealing the genomic composition of ST772-MRSA-V [19-23]. This lineage is closely related to clonal complex CC1 and harbours the relatively small and mobile staphylococcal cassette chromosome SCC*mec* type V [22]. The draft genomes accommodate a novel prophage Φ-IND772PVL, carrying the enterotoxin gene *sea* and an operon encoding Panton-Valentine Leukocidin (PVL), *lukS/F-PV* [21]. This potent combination of toxins on the same prophage has so far not been reported in other strains of *S. aureus* [21]. A heavily truncated *hlb-*converting phage is also present, which carries only *scn* (staphylococcal complement inhibitor) as part of the immune evasion cluster [21]. In addition, a variety of enterotoxins and superantigens have been detected in ST772-MRSA-V, including the variable presence of *sec* and *sel,* as well as the enterotoxin gene cluster *egc* [8,15,20]. Finally, several genes encoding antibiotic resistance determinants (e.g., against beta-lactams, aminoglycosides, fluoroquinolones, tetracyclines) and non-synonymous mutations in resistance-associated genes have been discovered, which correspond to previously identified multidrug resistant phenotypes of ST772-MRSA-V [7,15,20,24].

Despite these advances, the genomic location and context of resistance and virulence factors, which are frequently associated with mobile genetic elements (MGEs) [1,25], has not yet been unambiguously determined in ST772-MRSA-V [8,20]. Published draft genomes of ST772-MRSA-V have employed short-read sequencing technologies (i.e., Illumina), resulting in relatively fragmented assemblies (43–78 contigs, Table 1) and annotations and may lack important positional information of elements associated with resistance or virulence (e.g., resistance genes in [20]). Recently, third-generation sequencing technologies have allowed for the closure of complex genomic regions and the recovery of contiguous genome sequences [26,27], including for *S. aureus* [28-30].

**Table 1:** **Draft genomes of ST772-MRSA-V used for comparison with DAR4145**

**Strain**	**Origin**	**Year of isolation**	**Core genome SNPs vs. DAR4145**	**Number of contigs**	**Publication**	**Genbank accession**
**07-01748**	Western Australia	2007	72	70	Monecke et al. [20]	AZBT00000000
**KT/Y21**	Terrenganu, Malaysia	2009	73	70	Suhaili et al. [23]	AOCQ00000000
**118**	Bangalore, India	2008	70	78	Prabhakara et al. [21]	AJGE00000000
**120**	Bangalore, India	2009	52	56	Prabhakara et al. [21]	ALWE00000000
**333**	Madurai, India	2010	78	54	Prabhakara et al. [21]	ALWF00000000
**VH60**	Bangalore, India	2007	65	44	Prabhakara et al. [21]	ALWG00000000
**3989**	Hyderabad, India	2007	79	43	Prabhakara et al. [21]	ALWH00000000
**LVP2**	Bhubaneshwar, India	2010	119	73	Balakuntla et al. [22]	AOFV00000000
**3957**	Hyderabad, India	2007	254	43	Balakuntla et al. [22]	AOFU00000000

In this study, we present the complete genome sequence of DAR4145, a multidrug resistant strain of ST772-MRSA-V from Mumbai, India [31]. Pacific Bioscience (PacBio) single-molecule real-time (SMRT) sequencing resulted in a single contig containing the complete chromosome of DAR4145, which was error checked and corrected with Illumina reads. Annotation and comparison to nine other isolates from Australia, Malaysia and India highlighted an association of resistance and virulence determinants with MGEs. Additional resistance-associated mutations and gene redundancy explain the multidrug-resistant phenotype of DAR4145. The closed genome sequence fully defines the genomic composition of DAR4145 and provides a valuable reference genome for future investigations into the genomic epidemiology and phylogeography of ST772-MRSA-V.

## Results and discussion

**Figure 1:**
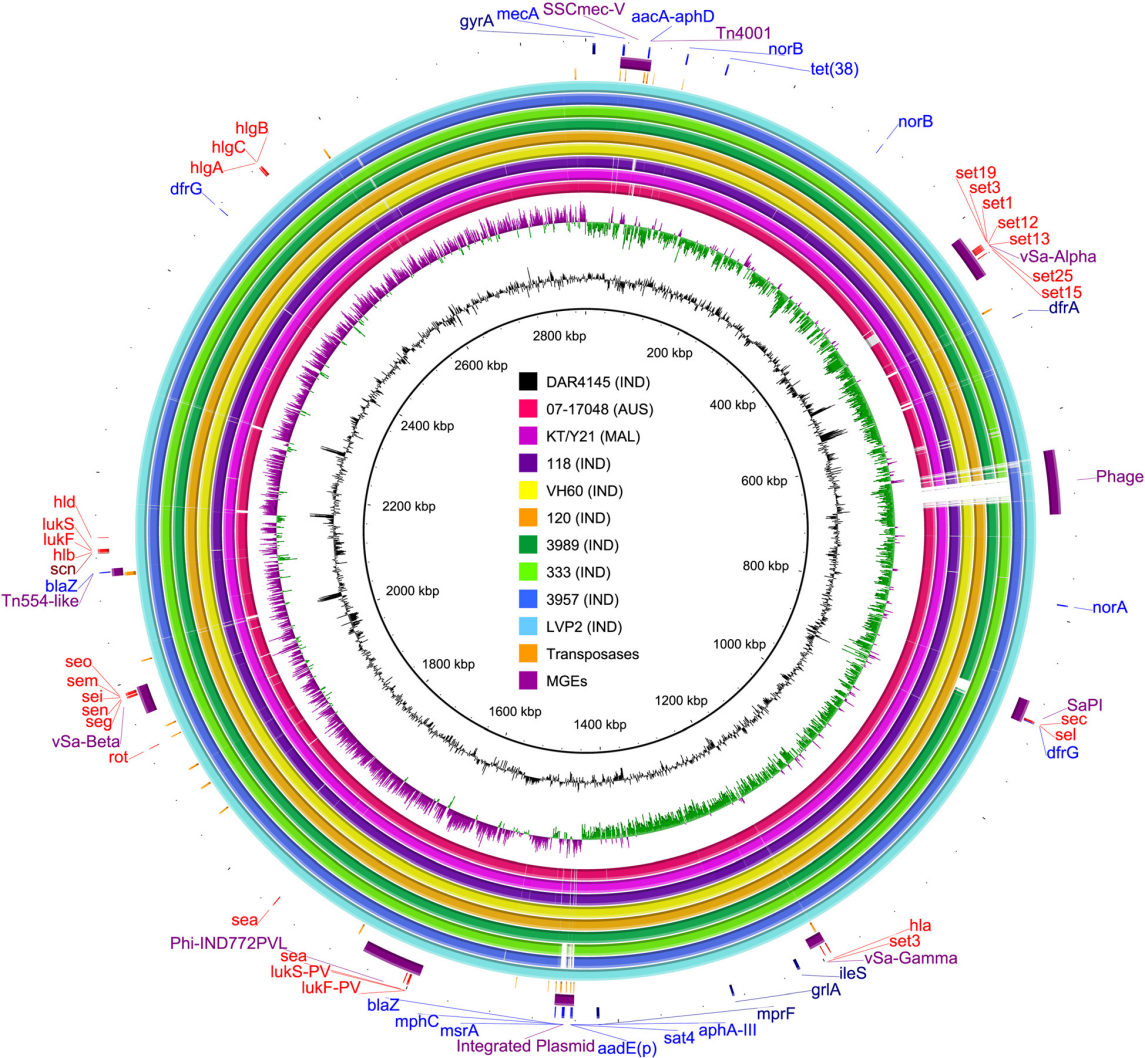
Comparison of draft genomes of nine strains of ST772-MRSA-V to the complete reference genome of strain DAR4145. The outer ring describes the location of selected genes associated with antibiotic resistance (blue), resistance mutations (dark blue) and selected staphylococcal toxins and superantigens (red) in DAR4145. The second ring denotes mobile genetic elements, including pathogenicity islands, phages and the integrated resistance plasmid, as well as the staphylocccal cassete chromosome SSC*mec*-V-C2 (purple). The third ring designates the location of transposases (orange) as determined by the final annotation of DAR4145. The subsequent rings and colours in the legend represent the strains used for comparison with DAR4145.The innermost rings shows the GC content (black) and GC skew (green -, purple +). Note that redundant genes in the reference genome (*dfrG*, *blaZ*) appear as multiple copies in the comparison genomes, even if they only exist as a single copy (due to comparison based on BLAST).

SMRT sequencing and subsequent assembly generated a single continuous sequence containing the complete chromosome of DAR4145 (Figure 1). A second shorter sequence was identical to the plasmid pKH-18, carrying cadmium resistance gene *cadD* and its regulator *cadX* [GenBank: EU333812.1]. The length of the completed chromosome was 2,860,508 base pairs (bp) with a G + C content of 32.85%. 2,642 protein coding regions, as well as 18 rRNAs, 61 tRNAs, 25 ncRNAs (including *RNAIII* and *sprD)* and 35 transposases associated with insertion sequence elements and transposons (including partial and putative sequences) were identified and annotated. Notably, gaps in the draft genome assemblies of other strains of ST772-MRSA-V (Figure 1) frequently occurred in the vicinity of transposases, suggesting that some of the short read assemblies were unable to bridge gaps associated with repetitive genomic elements. This clearly emphasizes the potential for obtaining fully defined *de novo* assemblies using third-generation sequencing technologies, which are able to resolve both local and global repeats [32] and avoid the introduction of reference bias. *In silico* multi-locus sequencing typing confirmed ST772, with a single allele divergence from ST1 (*pta*-22). Although a single locus variant of ST1 (and thus part of clonal complex 1), the *agr* locus of DAR4145 is part of *agr* group II rather than group III. A close relationship of the core genome is evident for all available genomes of ST772-MRSA-V. Excluding MGEs, the pairwise core genome SNP differences between DAR4145 and other ST772-MRSA-V genomes ranged from 52 to 254 SNPs (Table 1). DAR4145 harbours the staphylococcal cassette chromosome SCC*mec* type V containing the cassette recombinase *ccrC2*; the composition and arrangement are nearly identical to the previously identified SCC*mec* in strain 118 [22], with the exception of an additional mobile element inserted immediately upstream of the terminal direct repeat DR2. The element carried the bifunctional aminoglycoside modifying gene *aacA-aphD* and was flanked by transposases of IS256. Its composition identified the element as transposon Tn4001 [33] [GenBank: AB682805.1] and it was present at the same location in all available genomes of ST772-MRSA-V, except strain 118 (Figure 1).

### Resistance to multiple antibiotics mediated by mobile genetic elements

We found several additional resistance-associated genes, most of which were located on MGEs and correlated with the resistance phenotype of DAR4145 (Table 2). One core genome copy of the dihydrofolate reductase gene *dfrA* and two copies of *dfrG* were identified, which have been linked to high-level trimethoprim resistance in *S. aureus* [34]. The first copy of *dfrG* was found in the context of several phage genes and within the boundary of the terminal attachment site (*attR*) of a putatitve SaPI, partially identical with the pathogenicity island SaPITokyo12381 (BLASTn 99% identity, 70% coverage) [GenBank: AB860418.1]. The island also contained the enterotoxin genes *sec* and *sel,* placed in a similar context and position as *v*Sa3 Type II, a pathogenicity island of strain MW2 [35]. Monecke et al. [8] noted the frequent but variable presence of *sec* and *sel* in ST772-MRSA-V, which was supported in this study by the absence of the putative SaPI in strain 3989 (Figure 1). The second copy of *dfrG* was found in the chromosome at 2,474,700 bp. Both sequences, with two adjacent coding regions for hypothetical proteins, were identical to genomic regions in ST239 strain TW20 from the UK [36], the trimethoprim-resistant *S. pyogenes* strain A1085 from India [37] and part of the transposon Tn6198 from *Listeria monocytogenes* [38] [GenBank: FN433596.1, GenBank: JX498941.1, GenBank: JX120102.1]. Analysis with ResFinder [39] confirmed the presence of the two *dfrG* copies in DAR4145, but only identified the SaPI-associated copy in the draft genomes of 07–17048, 118, 3957 and KT/Y21 and the core-associated copy in isolates 120, 333, 3989, LVP2, VH60. The presence of *dfrG* was also confirmed by mapping paired-end short reads against our resistome database and corresponded to a high-level trimethoprim/sulfamethoxazole (TMP-SXT) resistance phenotype of DAR4145 (MIC ≥ 320 μg/L) (Table 2). The *dfrG* gene was previously thought to be rare in *S. aureus* colonising humans, but has recently been found to be widespread in sub-Saharan Africa [34].

**Table 2:** **Selected phenotypic and genomic antibiotic resistance profiles in strains of ST772-MRSA-V**

**Antibiotic**	**Phenotype (MIC μg/L)**		**Resistance Genes and Mutations in ST772-MRSA-V**									
	**DAR4145**		**DAR4145**	**07-17048**	**KT/Y21**	**118**	**120**	**333**	**3957**	**3989**	**LVP2**	**VH60**
Oxacillin	R (≥4)	*mecA* **(M)**	+	+	+	+	+	+	+	+	+	+
		*blaZ * ***(M)***	+	+	+	+	+	+	+	+	+	+
		*blaZ * ***(P)***	+	-	+	+	+/−	-	-	+	+	+
Gentamicin	R (≥16)	*aacA-aphD* **(M)**	+	+	+	-	+	+	+	+	+	+
		*aphA-III, sat4, aadE(p)* **(P)**	+	+	+	+/−	+	-	-	+	+	+
Trimethoprim/Sulfamethoxazole	R (≥320)	*dfrA* **(C)**	+	+	+	+	+	+	+	+	+	+
		*dfrG* **(C)**	+	-	-	-	+	+	-	+	+	+
		*dfrG* **(M)**	+	+	+	+	-	-	+	-	-	-
Ciprofloxacin Levofloxacin Moxifloxacin	R (4) R (4) I (1)	*norA/norB* **(C)**	+	+	+	+	+	+	+	+	+	+
		*grlA* **(S80T/F)**	+	+	+	+	+	+	+	+	+	+
		*gyrA* **(S84L)**	+	+	+	+	+	+	+	+	+	+
Tetracyline	S (≤1)	*tet(38*) **(C)**	+	+	+	+	+	+	+	+	+	+
Erythromicin Clindamycin	I (1) S ( ≤0.25)	*msrA-mphC* **(P)**	+	+	+	+	+	-	-	+	+	+

(R = Resistant, I = Intermediate, S = Susceptible,C = Chromosomal, M = Mobile Genetic Element, P = Integrated Resistance Plasmid, + = present, − = absent, p = partial, +/− = partial or truncated).

Similarly, two copies of the beta-lactam resistance operon (*blaZ, blaI, blaR)* were identified in DAR4145. The first copy was part of a partial integrated plasmid highly similar in composition to the plasmid p18810-03 (BLASTn 99% identity, 85% coverage) [GenBank: CP002141.1] found in CA-MRSA strain 18810 of USA300 [40] and nearly identical with the integrated plasmid in strain 11819–97 of CA-MRSA ST80-IV (BLASTn 99% identity, 99% coverage) [GenBank: CP003194.1] [28]. The integrated plasmid encoded a resistance cluster composed of *aphA-III, sat4* and a partial *aadE* (aminoglycoside resistance), as well as *mphC* and *msrA* (macrolide resistance), thus resolving the genomic context of these genes previously noted by Monecke et al. [20]. Draft genomes of strains 07–17048 and 120 respectively lacked or carried a truncated, plasmid-associated *blaZ*, whereas the integrated plasmid was entirely absent in strains 333 and 3957 (Figure 1, Table 2). The second copy of the beta-lactam resistance operon was located immediately downstream of three transposases related to Tn554 and was present in all isolates of ST772-MRSA-V. The Tn554-like transposon [41] was previously found with nearly identical sequence composition in H-EMRSA-15 [CP007659.1] and HO 5096 0412 [GenBank: HE681097.1] of ST22 [41,42].

### Resistance to multiple antibiotics mediated by core chromosome genes and mutations

Three additional, chromosomal resistance genes identified in DAR4145 were the transporter genes *norA* and *norB*, whose altered expression is associated with quinolone resistance [43,44] and the efflux pump *tet*(38)*,* which can confer resistance to tetracyclines when upregulated [43,45,46]. The presence of *tet*(38) alone did not result in phenotypic resistance for DAR4145 (Table 2). Notably, *tet*(38) also facilitates the efflux of antibacterial fatty acids, which promotes survival in an abscess environment and colonisation of skin surfaces [46].

Furthermore, the chromosomal genes *grlA*/*grlB* and *gyrA*/*gyrB* harbour common non-synonymous mutations associated with increased resistance against quinolones in *S. aureus* [47-49]. We found two mutations in ST772-MRSA-V, one in *grlA* (S80Y, S80F in isolates 3957 and LVP2) and another in *gyrA* (S84L, in all except isolate 118). These correspond to ciprofloxacin-resistance associated mutations previously found in ST772-MRSA-V and other STs from India [24]. We also detected non-synonymous mutations in the genes *dfrA* (V134I), *ileS-1* (D621H) and *mprF* (L335S) that were present in all examined strains of ST772-MRSA-V. These were located in the vicinity of mutations that have previously been linked with resistance to trimethoprim [50], mupirocin [51] and daptomycin [34] in *S. aureus*, respectively.

### Phage and virulence determinants

In addition to the antibiotic resistance factors and the putative SaPI, DAR4145 harbours the genomic islands *v*Saα, *v*Saβ and *v*Saγ. These were present in all investigated genomes of ST772-MRSA-V (Figure 1) and encoded several superantigen-like proteins and toxin determinants, including the enterotoxin gene cluster (*egc*) on *v*Saβ as well as α-haemolysin (*hla*) and exfoliative toxin A (*eta)* on *v*Saγ. In addition, *v*Saα harbors *aadK*, encoding aminoglycoside 6-adenylyltransferase, which is associated with low-level streptomycin resistance in *Bacillus subtilis* [52]. The recently described prophage Φ-IND772PVL, containing the staphylococcal enterotoxin A (*sea*) and the PVL-operon (*lukF-PV, lukS-PV)*, was also found in all isolates, confirming its widespread presence in ST772-MRSA-V from India [21]. DAR4145 harboured the heavily truncated *hlb-*converting prophage encoding the staphylococcal complement inhibitor (*scn*) [20,21], immediately followed by the *hlg*-related leukocidin genes *lukF* and *lukS* [53]. We also found a second putative enterotoxin gene with high similarity to *sea* within the chromosome at 1,746,983 bp (BLASTn, 98% identity, 98% coverage in CA-MRSA ST80-IV strain 11819–97) [GenBank CP003194.1]. Additionally, DAR4145 contained one previously undetected prophage region at 637,316 bp. This prophage did not harbour known resistance or virulence genes and the central region was present only in DAR4145, 3957 and LVP2 (Figure 1). The entire region was similar to φMu50B of strain Mu50 (BLASTn, 95% identity, 83% of total coverage) [GenBank: BA000017.4], with the core region showing highest similarity to φNM1 and φNM2 (BLASTn, 96% identity, 51-54% of total coverage) [GenBank: DQ530359.1 and DQ530360.1].

Finally, a putative and a truncated transposase for IS1272 were found in the vicinity (−477 bp) of the global transcriptional regulator of virulence *rot* (repressor of toxins). Benson et al. [54] demonstrated that insertion of IS257 elements into the promoter region of *rot* was responsible for derepression of cytotoxin expression and increased virulence of USA500 strains. Although we did not examine the functional significance of this arrangement, the role of gene expression regulation is becoming increasingly recognized to contribute to variations in virulence and antibiotic resistance of *S. aureus* [43,46,54,55] and thus may be of interest to future investigations into ST772-MRSA-V. Similarly, the functional role of the *agr* group II locus in DAR4145 is of interest as it differs from the *agr* group III locus found in other clonal complex 1 strains [56].

### Comparison to other STs

There are notable differences between DAR4145 and other sequenced CA-MRSA strains with regards to acquired antimicrobial resistance and virulence determinants (Additional file 1). The level of multi-drug resistance is greater than that seen in the key CA-MRSA clones from the US – USA300 [57] and MW2 [35], Europe – ST80 [28], Australia – ST93 [58], and Asia – ST59 [59]. In particular, DAR4145 has MGEs encoding for resistance to cotrimoxazole and aminoglycosides that are not present in the other CA-MRSA genomes. The integrated plasmid harbouring resistance genes *blaZ*, *mphC*, *aphA-III* and *msrA* is found only in DAR4145 and ST80 strain 11819–97; although in contrast to 11819–97 the integrated plasmid is not within the *SCCmec* element in DAR4145 [28].

Although all the above listed CA-MRSA strains harbor PVL-encoding phage, the combination of *sea* and PVL on Φ-IND772PVL only occurs in ST772-MRSA-V [21]. In comparison to the other strains that carry 2–7 enterotoxin genes, DAR4145 harbors 10 such genes, including *sec* and *sel* on the putative SaPI and the enterotoxin cluster *seg, sei, sem, sen* and *seo* on *v*Saβ. DAR4145 contains capsid type 5, but lacks both the serine-like protease operon *spl* and *lukD/E*. In total, compared to other important genome sequenced CA-MRSA strains, DAR4145 contains a striking combination of acquired antimicrobial and virulence determinants. It should be noted that the small number of available genomes examined in this study may cause a bias towards isolates that are particularly virulent or multi-drug resistant and therefore may not be an accurate assessment of these traits in the lineage at large. However, previous studies on larger collections that included molecular typing of resistance and virulence factors have determined similar resistance profiles and confirmed the widespread presence of virulence determinants, such as the *egc* gene cluster or PVL [7,15,21].

## Conclusions

The complete genome of ST772-MRSA-V is remarkable for the presence of multiple MGEs mediating a highly antibiotic resistant phenotype. This phenotype is further augmented by the presence of core genome resistance-associated mutations and redundancy of some resistance mediating genes. For example, DAR4145 demonstrates high-level resistance to TMP-SXT (MIC ≥ 320 μg/L) and contains three relevant resistance genes, *dfrA* and two copies of *dfrG*. Additionally, DAR4145 carries a large complement of virulence factors including multiple enterotoxins, α-haemolysin and PVL.

Recommended oral antibiotics for the treatment of community-associated *S. aureus* skin and soft tissue infections include anti-staphylococcal β-lactams, clindamycin, TMP-SXT, erythromycin, and doxycycline [60]. DAR4145 is resistant to all of these antibiotics except for doxycycline (and even then it carries *tet*(38), which if up-regulated could conceivably result in phenotypic resistance). Unfortunately, a multidrug-resistant genotype is not restricted to DAR4145 and appears to be a consistent feature of all ST772-MRSA-V strains analysed in this study. Thus, simple oral treatment options for the ST772 lineage are truly limited.

The increasing prevalence of ST772-MRSA-V in India and its appearance in multiple regions of the world [7-9,12-14,20], should raise concerns. As with highly resistant Gram-negatives, drivers of the emergence and spread of ST772-MRSA-V need urgent study. Here, we have defined the bacterial factors that contribute to the multidrug-resistant phenotype of this lineage. Studies to understand the global phylogeography of ST772 can now make use of the closed reference genome of DAR4145.

## Methods

### Sequencing and assembly

Strain DAR4145 was recovered as a blood culture isolate in a patient from Mumbai, India in 2009 [31]. This strain was multilocus sequence type 772, *spa* type 1080 (Ridom *spa* type t657), PVL+, SCC*mec* V, and *dru* type 10bk. For this study, antimicrobial susceptibility testing was performed with the Vitek2 platform using 22359 VITEK AST-P612 cards (bioMerieux, France) with Clinical and Laboratory and Standards Institute breakpoints utilised [61].

Sequencing for *de novo* assembly was performed on the PacBio RS (Menlo Park, CA, USA). A sequencing library (SMRTbell) was generated from approximately 2 ug of genomic DNA sheared to an average fragment length of 10 kb. Library preparation was carried out following manufacturer protocols using DNA Template Prep Kit 2.0 (3 – 10 Kb). The sequencing enzyme used was version 2.0 from the DNA/Polymerase Binding Kit 2.0, movie lengths were 90 min, and sequencing chemistry was version C2. From eight SMRT cells of sequencing, we generated 313,192 polymerase reads with an average length of 3.1 kb (and a total of 401,221 sub-reads with an average length of 2.0 kb, the largest was 14.7 kb; sub-reads are the individual forward and reverse reads of every SMRTbell library molecule excised from a polymerase read). *De novo* assembly was performed with HGAP v.2.2. Circularisation was achieved by manual comparison and removal of a region of overlap. For short-read re-mapping and error-correction, an Illumina library with a median insert size of 238 bp (first and third quartile: 194/303 bp) was generated and sequenced on the Illumina HiSeq 2000 with 100 bp paired-end runs, producing 3,826,612 sequencing reads.

### Computational analysis

Automated error correction was performed by mapping paired-end short reads against the PacBio assembly with five iterations in iCORN v.2 [62]. Results were confirmed with SPANDx v.2.4, using standard parameters [63]. The corrected reference sequence was annotated with Prokka v1.07 [64] and supplemented with results from the annotation servers BASys [65] and RAST [66]. Visual inspection and manual curation was carried out with Artemis v.16.0.0 [67,68] and BLAST searches against databases from NCBI [69]. Virulence factors and phages were identified using VirulenceFinder 1.2 (90% identity) [70] and the PHAST server [71]. *In silico* multi-locus sequence typing (MLST) was performed using the MLST server v1.3 [72] and validated by mapping paired-end short reads to a previously assembled MLST database from Sanger Institute.

We obtained publicly available assemblies and short reads for nine additional whole-genome sequences of ST772-MRSA-V (07–17048, 118, 120, 333, 3957, 3989, VH60, LVP2, KT/Y21; see Table 1) for comparison and visualization with the BLAST Ring Image Generator (BRIGS) v.0.95 [73]. Each genome was also mapped against DAR4145 using SMALT v.0.7.5 with default settings, followed by exclusion of regions of known MGEs. High-quality core genome SNPs were identified using a combination of SAMtools v.1.1 mpileup and bcftools, with filters applied as previously described [74]. Genes and high-quality SNPs implicated with antimicrobial resistance were identified in all strains by mapping available short read data against a database of known, annotated sequences from Sanger Institute with SMALT v.0.7.5 (Sanger Institute) and by using an in-house pipeline combining GATK v.3.3.0 [75,76] and SAMtools v.1.1 [77,78]. Resistance genes were verified with ResFinder v.2.1 [39] at 80% minimum length and 90% identity cutoffs, using assemblies ordered against the reference DAR4145 in Mauve v.2.3.1 [79] in order to determine their location and genomic context. Specific regions of interest (i.e., resistance genes, phages, virulence factors) were further investigated and validated by mapping available short reads of isolates, including DAR4145, against the PacBio assembly with standard parameters in SPANDx. Finally, we obtained representative genomes of five significant STs for comparison with ST772-MRSA-V in ResFinder and Virulence Finder: MW2 [GenBank: BA000033.2], USA300 FPR3757 [GenBank: CP000255.1], ST59 M013 [GenBank: CP0003166.1], ST80 11819–97 [GenBank: CP003194.1] and ST93 JKD6159 [GenBank: CP002114.2]. Results were visualized as a presence/absence matrix in MS Excel 2010.

### Ethics statement

The bacterial isolate DAR4145 was collected as part of routine clinical service provision [31]. The use of a de-identified bacterial strain described in this manuscript, and the study of the bacterial isolates and not human subjects, meant that formal Human Ethics Committee approval or Informed Patient Consent was not required.

### Availability of supporting data

The complete genome assembly and annotation of DAR4145 has been submitted to GenBank under the accession number CP010526. Illumina reads are available at the European Nucleotide Archive under the accession number ERS161279.

## Additional file

Additional file 1:
**Comparison of resistance and virulence genes in representative genomes of five significant STs and ST772-MRSA-V DAR4145.**

## Abbreviations

MGE: Mobile genetic element
MLST/ST: Multilocus sequence type/sequence type
(CA-/HA-) MRSA: (community-associated-/healthcare-associated) methicillin-resistant Staphylococcus aureus
PacBio: Pacific biosciences
SaPI: Staphylococcus aureus Pathogenicity Island
SMRT: Single-molecule real-time sequencing
TMP-SXT: Trimethoprim/sulfamethoxazole
